# A prospective, comparative study of the analgesic effect between the WALANT technique and local anesthesia associated with sedation for hand surgery

**DOI:** 10.1590/acb384323

**Published:** 2023-10-30

**Authors:** Patrick Rech Ramos, Rioko Kimiko Sakata, Helio Costa Ribeiro, Aline Bonfanti, Leonardo Henrique da Cunha Ferraro

**Affiliations:** 1 Universidade Federal de São Paulo – Ciência Cirúrgica Interdisciplinar Posgraduation Program – São Paulo (SP) – Brazil.; 2 Universidade Federal de São Paulo – Pain Sector – São Paulo (SP) – Brazil.; 3 Hospital Santa Terezinha – Erechim (RS) – Brazil.

**Keywords:** Hand, Epinephrine, Lidocaine, Anesthesia, Local

## Abstract

**Purpose:**

The primary objective of this study was to compare the WALANT (wide awake, local anesthesia, no tourniquet) technique with local anesthesia associated with sedation in relation to pain intensity for minor hand surgical procedures. The secondary objective was to evaluate the need for analgesic complementation.

**Methods:**

A prospective, randomized, comparative, and clinical study was carried out. The sample size in each group was determined after statistical evaluation of the results of a pilot project. The participants were allocated to one of two groups; those in group 1 were submitted to the WALANT technique, and those in group 2, to local anesthesia associated with sedation, for elective surgery. The surgical procedures were carpal tunnel syndrome, De Quervain’s tenosynovitis, synovial cyst, finger cyst, and trigger finger. Pain intensity, need for complementation and evolution to complex regional pain syndrome were evaluated.

**Results:**

There was no difference between groups in pain intensity after WALANT and need for intraoperative complementation. There was no significant difference in the amount of opioid applied postoperatively between the groups. There was no difference between groups regarding comfort during surgery. There was no difference in adverse effects and complications between the groups. Hematoma was the most frequent adverse event. No severe adverse events were observed.

**Conclusions:**

The WALANT technique promoted an analgesic effect similar to that of local anesthesia associated with sedation, without increasing adverse effects.

## Introduction

In the past, local anesthetics associated with a vasoconstrictor should not be administered to extremities because it causes ischemia, with the worst event being digit necrosis. In a review of the literature from 1880 to 2000, one author noted that most reported cases of finger necrosis were not associated with the use of adrenaline^1^. After this study, the Dalhousie University project started in Canada to evaluate the use of local anesthetic with vasoconstrictor for hand surgery^2^.

Minor surgical hand procedures are usually performed under local anesthesia and sedation^3^ and hemostasis with a tourniquet. Since the advent of the WALANT (wide awake, local anesthesia, no tourniquet) technique, with studies proving its safety, a migration to this technique by surgeons for minor hand operations has been observed^4^. In the WALANT technique, no tourniquet is used, which is tolerated for 15 to 20 minutes^5^. Tourniquet can also cause complications such as pain, nerve and/or muscle injury, and skin necrosis, depending on the degree of compression, length of maintenance, and clinical conditions of the patient^6^.

In the WALANT technique, the surgery is performed with the patient awake and injection of solution with 9 mL of 1% lidocaine with adrenaline and 1 mL of 8.4% sodium bicarbonate^7^, without using a tourniquet. The operation is performed in any setting as an in-office procedure or in the operating room^8^. This technique allows the surgeon to interact with the patient and even request that the patient actively participate during the operation. This interaction is important especially in surgeries such as those for extensor or flexor tendon injury or trigger finger. Despite this, no studies on the analgesic efficacy of this technique have been found that can relate to the Brazilian population. This study can provide data on the benefit of the technique without harming the patients.

The primary objective of this study was to compare the WALANT technique with the local anesthesia technique associated with sedation regarding pain intensity for minor hand surgical procedures. The secondary objective was to compare the need for complementary analgesics.

## Methods

A prospective, randomized, and comparative study was conducted.

The study was conducted after approval by the Ethics Committee (CAAE 34666920.3.0000.5505), and the patients’ signing of the informed consent form. All research was based on Resolution no. 466/12 of the National Health Council, which defines the guidelines and standards for research involving human subjects. The study was carried out according to the Consolidated Standards of Reporting Trials (CONSORT)^9^.

The allocation of the participant to one of the two groups was as such. We used www.random.org to allocate numbers to patients and the group they would join. We had patients from 1 to 56 and groups 1 and 2. When a patient was scheduled to surgery, the hospital’s secretary, which was responsible for booking the surgery, would assign a ballot to the patient, that number would be from 1 to 56. Then, in a sheet she would see what group that number referred to. For example, the first patient to enroll the study could have been drawn the ballot number 42, which by random.org randomization would be group 2, for instance, and so on.

To determine the sample calculation, a pilot project was conducted with 10 participants in each group, and the necessary information was obtained from it. Then, type error up to 0.05, and type-II error of 0.2 were applied, which results in study power of 80%, for a comparison test between independent groups, based on normal distributions. The pain score of 0 to 10 was employed for primary outcome, with the aim of identifying a minimum clinically significant difference of 2. Data from the pilot study of 10 participants were used to identify variability in the sample data, which identified a standard deviation of 2.7. Thus, it was identified that a sample size of 28 individuals in each group would be necessary to achieve a study power of 80%.

The study included patients: 18 years of age or older; of either gender; ASA I or II, by the American Association of Anesthesiologists; undergoing minor hand surgery (carpal tunnel syndrome, wrist, and finger synovial cysts, De Quervain’s tenosynovitis, and trigger finger) confirmed by physical examination or complementary tests (ultrasonography, electroneuromyography); ambulatory; operated on by a physician specialized in hand surgery. The following patients were excluded: cognitively impaired (unable to understand the study); psychiatric illness; vasospastic disorder; thromboangiitis obliterans; infection at the puncture site; drug allergy; previous surgery; use of anticoagulants; and pregnant.

WALANT group received lidocaine with epinephrine and sodium bicarbonate. In the procedure room out of the operating room, after antisepsis, the patient underwent local infiltration with 9 mL of 1% lidocaine with adrenaline 1:100,000 associated with 1 mL of 8.4% sodium bicarbonate according to the literature^10^. After anesthesia, 25 minutes were waited until the hemostatic effect of adrenaline. Electrocautery would be used only if there was major bleeding, and if it could not be contained, preventing surgery, pneumatic tourniquet could be used. If complementary anesthesia was needed during surgery, lidocaine infiltration at 1% without adrenaline could be performed, not exceeding 7 mg/kg. After the completion of the surgery, a dressing was applied, and the patient was discharged from the hospital. Local anesthesia group received lidocaine without epinephrine associated with sedation.

In the operating room, after monitoring the patient and obtaining venous access, propofol bolus and infusion were administered by the anesthesiologist. After local anesthesia made by the surgeon with 10 mL of lidocaine 1%, without adrenaline, the upper limb was exsanguinated with an Esmarch strip, and another strip placed near the axilla to keep the limb exsanguinated. Afterwards, a pneumatic tourniquet was inflated to 250 mmHg, and the surgery was performed without the use of electrocautery, followed by dressing and removal of the tourniquet. The patient was moved to the anesthesia recovery room, pending hospital discharge criteria: lucid, oriented patient, no pain, no nausea, stable vital signs, no signs of bleeding at the surgical site^11^.

At discharge, patient was instructed to use dipyrone 1 g if pain ≥ 4 to 6 times a day. If pain persisted ≥ 4 times after dipyrone, the patient could take tramadol (50 mg) up to 400 mg a day. The patient was required to record the intensity of pain before medication administration.

Patients were assessed for: need for complementary intraoperative analgesia, pain intensity by verbal numeric scale after seven days, 14 days and one month, patient comfort during surgery^12^, postoperative analgesic complementation and Budapest criteria^13^ after one month. Adverse effects and complications were also recorded.

The primary outcome was a reduction in postoperative pain intensity. Secondary outcomes were a reduction in the need for analgesics and in the progression to complex regional pain syndrome.

For statistical analysis, categorical data were described by their absolute occurrence and their relative frequency between groups, and the comparison between groups was performed by Pearson’s or Spearman’s chi-square test. Continuous data were tested for their distribution, Shapiro-Wilk’s test, and described by the mean and standard deviation. Student’s t-tests were used for pain intensity in the first week and age, the Mann-Whitney’s test for weight, height, duration of surgery, and pain intensity in the second week and one month, and the chi-square test for gender and complications/adverse effects. A p ≤ 0.05 was considered significant.

## Results

**Figure 1:**
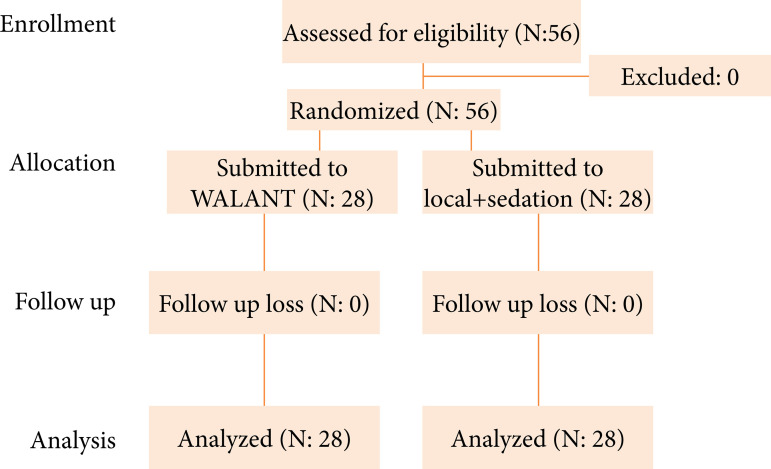
CONSORT flow chart.

Source: Elaborated by the authors.

The CONSORT flowchart is shown in Fig. 1. Fifty-six participants were evaluated for eligibility, and were randomized. There was no loss of participants, with 56 being analyzed. Participants’ data are shown in Table 1. There was no difference in the duration of surgery between groups (WALANT: 10.9 + 0.9 minute; local + sedation: 9.5 + 1.3 minute, Mann-Whitney’s test; p = 0.657). There was no need for complementary local anesthetic during surgery for any patient. There was no difference in pain intensity between groups (Table 2). There was no difference in the need for postoperative analgesic complementation (Table 3). There was no difference in comfort during surgery between groups (WALANT = 9.5 + 1.3; local + sedation = 9.71 + 1; Mann-Whitney’s test; p = 0.314). There was no difference in adverse effects and complications between the groups (Table 4). The most frequent adverse event was hematoma (26 occurrences, of them in G1 = 39.3%; G2 = 35.7%) (Table 4). No serious adverse events were observed.

**Table 1:** Participants’ data. Mean ± standard deviation for age, weight, height, and number for gender.

	WALANT	Local + sedation	p-value
Gender (F / M)	21:7	24:4	0.313£
Age (years)	51.8 ± 9.6	51.6 ± 14.0	0.956!!
Weigh (kg)	70.3 ± 8.8	68.5 ± 7.8	0.432§
Height (cm)	1.6 ± 0.9	1.6 ± 0.1	0.558§
Duration of surgery (min)	10.9 ± 0.9	11.9 ± 0.9	0.657§

§ Mann-Whitney’s test;

!! student’s test;

£ chi-square test.

Source: Elaborated by the authors.

**Table 2:** Intensity of pain (maximum) by numerical scale. Mean ± standard deviation.

	WALANT	Local + sedation	p-value
T0	0	0	NC
1 week	3.0 ± 2	3.2 ± 2.7	0.699!!
2 weeeks	1.2 ± 1.9	1.4 ± 2.3	0.799§
1 month	0.5 ± 1.6	0.6 ± 1.4	0.795§
Comfort during surgery	9.5 ± 1.3	9.7 ± 1.0	0.314§

§ Mann-Whitney’s test;

!! student’s test; NC: not calculated.

Source: Elaborated by the authors.

**Table 3:** Complementary analgesics: number of patients (%).

		WALANT (%)	Local + sedation (%)	p-value
Dipyrone				
	1 week	28 (100)	27 (96.42)	0.528
	2 weeks	8 (28.57)	10 (35.71)	
	1 month	1 (3.57)	2 (7.14)	
Tramadol				
	1 week	2 (7.14)	3 (10.71)	0.561
	2 weeks	1 (3.57)	2 (7.14)	
	1 month	0	0	

Chi-square test. Source: Elaborated by the authors.

**Table 4:** Side effects and complications: number (%).

	WALANT	Local + sedation	p-value
Hematoma	11 (39.3)	10 (35.7)	
Edema	4 (14.3)	0 (0)	
Nausea	0 (0)	3 (10.1)	
Paresthesia	1 (3.5)	1 (3.5)	
Dehiscence	0 (39.3)	1 (3.5)	0.368
Infection	0	0	
TOTAL	16	15	

Chi-square test. Source: Elaborated by the authors.

## Discussion

The infiltration with local anesthetic with vasoconstrictor for hand surgery promoted analgesic effect similar to local anesthesia associated with sedation, without the increase in adverse effects and complications. In this study, all patients were evaluated preoperatively and operated on by the same surgeon, a board certified hand surgeon.

The local anesthetic is acidic, causing pain during its administration, and the bicarbonate has the action of neutralizing the acidity, decreasing considerably the pain of the injection. The pain is mild to none as long as the infiltration is applied slowly and with pauses.

Patients could feel discomfort when undergoing surgery without sedation, however, on the questionnaire, they reported no discomfort from being awake, and comfort during surgery was similar regardless of the anesthetic technique^14^ performed on 24 patients with bilateral carpal tunnel syndrome, one side by the conventional technique and the other one by WALANT and reported that WALANT offered a better intraoperative experience than the conventional technique. In another study, satisfaction was higher in the WALANT group^15^, as well as in a review in which WALANT had the highest satisfaction rate^16^.

There was no further need for anesthetic or analgesic complementation after the procedure in both groups. The volume of local anesthetic (10 mL) is in agreement with the literature as sufficient to perform all proposed surgeries^17^^,^ 18. This study was conducted for minor hand surgery, which can be done with small amounts of local anesthetic. For larger surgeries, we need larger volumes, and because of this larger volume of anesthetic we must be aware of its toxicity.

In surgery with local anesthetic associated with sedation, bleeding is not observed through the use of pneumatic tourniquet. The WALANT technique must respect some principles, one of them is to wait for the vasoconstrictor to take effect before starting the surgery. According to one study, this time is between 25 to 30 minutes^5^. The authors of another study reported that 7 minutes of waiting is adequate^19^. More studies must be conducted to better evaluate. In this study, there was no excessive bleeding or need for electro-cautery, respecting the time of 25 minutes until skin incision.

In this study, the surgery was of short length, decreasing the time of tourniquet and ischemia of the sedation group. Also, the time to discomfort of the patient undergoing surgery without sedation was mild. In this study, the duration of surgery was not statistically significant, but it was longer in the WALANT group by a small margin. In a systematic review, the duration of surgical time was longer^16^. That refers to bleeding that occurs during surgery, even a small amount, causing a longer surgery time.

In this study, 25 minutes were waited before the surgical incision in the WALANT group, as in the literature^5^. In the sedated patient, there is no need for this waiting time, but there is the waiting time for venipuncture and sedation to be done before local anesthesia and upper limb exsanguination.

The duration of WALANT surgery in this research was slightly longer as in a systematic review^16^. Because no tourniquet was used during surgery, bleeding was observed, even if little, causing a longer surgery time. Even if one waits the recommended time between the infiltration and the incision, the surgical field is not completely free of bleeding as in surgeries performed with sedation and a tourniquet, which may cause a small increase in the duration of surgery. This is when only the time to perform the surgery is considered. Between the time from entering the operating room and hospital discharge, patients who have been sedated stay longer in the hospital, due to the need for recovery after sedation.

The intensity of postoperative pain in this study, with no difference between the groups, and the anesthesia used did not interfere with postoperative pain control, and opioid use was similar for conventional anesthesia and WALANT technique as in a study in the literature^20^. In another study, opioid use was more dependent on age and gender than the anesthetic technique employed^21^. In a systematic review, postoperative pain intensity was lower in the WALANT group^16^. Nevertheless, the surgical procedures in this study caused mild pain, with less need for opioids and good management with non-opioid medication.

In this study, the most frequent adverse event was hematoma, but with no difference between the groups. Hematoma can be related to the lack of tourniquet in the WALANT technique, but we haven’t found any reports in which the end of the epinephrin effect could cause an increase in bleeding. We haven’t found any reports suggesting the end of the use of tourniquet caused an increase in bleeding and hematoma neither, even though the sedation group had similar cases of hematoma as the WALANT group. In one review, the authors reported no difference in patient complications^16^.

The adverse effects in this study were similar in the groups, and no serious adverse events such as ischemia associated with vasoconstrictor use were observed. After hand surgery, it is a frequent occurrence to develop complex regional pain syndrome, impairing rehabilitation. The Budapest criteria is used for the clinical diagnosis of complex regional pain syndrome^13^. It was performed on all patients at one month follow-up. It requires at least one symptom in all symptom categories and at least one sign in two or more categories. No patient displayed criteria for the syndrome.

The comparison between adrenaline and non-adrenaline anesthesia without sedation cannot be done. Even though the expected surgery time for all diseases in the study is short, the non-adrenaline group need to exsanguinate the upper limb for surgery to be done. The exsanguination is painful for the non-sedated patient, and if surgery takes longer, the patient will feel a lot of discomfort. If exsanguination is not done, the surgery time of the non-adrenaline group would rise and with that the possibility of more complications, such as infection, hematoma and possibly others.

One advantage of the WALANT technique is that it can be done as in office procedure, without the need of a complete surgical setting. Another one is that the patient is discharged right after surgery, which can potentially decrease cost, and since sedation is not performed the patient does not need to be fasting. One disadvantage is the possibility of vasovagal reflex, that is easily reversed by stopping the local anesthesia and elevating the patient legs until they feel better.

As limitations of the study, it is not possible to blind the study completely. The sample was small compared to other similar studies, and, even though all illnesses had similarities with the surgery done, it is hard to compare different diseases that can have different outcomes postoperatively. A larger study with only one disease comparing the two techniques would be necessary.

## Conclusion

The WALANT technique promoted an analgesic effect, patient comfort and the necessity for complementation similar to that of local anesthesia associated with sedation, without increasing the adverse effects for hand surgeries.

WALANT is the authors’ preferred technique for minor hand surgeries.

## Data Availability

The research data can be found in the reservoir DOI: https://doi.org/10.17632/4hf9s549b5.1
